# Insights from an extensive multi-step multi-stakeholder engagement in circular bio-based economy

**DOI:** 10.12688/openreseurope.21512.1

**Published:** 2025-12-17

**Authors:** Gülşah Yilan, Elina Dace, Mariam Rasheed, Luana Ladu, Valentina Vavassori, Susanna Albertini, Gabor Mester, Piergiuseppe Morone

**Affiliations:** 1 University of Rome Unitelma Sapienza, Rome, Italy; 2 Baltic Studies Centre, Riga, Latvia; 3 BAM Federal Institute for Materials Research and Testing, Berlin, Germany; 4 Technische Universitat Berlin, Berlin, Germany; 5 FVA New Media Research, Rome, Italy; 6 PEDAL Consulting s.r.o., Martin, Slovakia; 7 Corvinus Institute for Advanced Studies (CIAS), Corvinus University of Budapest, Budapest, Hungary

**Keywords:** Barrier, circular bio-based economy, focus group, sustainable transition, quadruple helix, validation.

## Abstract

**Background:**

The goal of achieving a net-zero emissions European economy by 2050 requires a shift from linear fossil-based to circular bio-based systems. However, this transition relies on societal transformations, cutting-edge technologies, and multi-actor processes, which in turn require a new economic framework and policy priorities. This study bridges the gap between literature and circular bio-based economy (CBBE) practice by presenting the results of stakeholder engagement activities where the barriers identified in the literature were discussed, ranked and validated.

**Methods:**

Adopting a three-step approach, including a focus group, interviews, and a final survey, we present a list of 31 barriers under six different clusters: cultural, economic, environmental, governance, structural, and technical hindering the transition to a CBBE.

**Results:**

The findings underline that the transition requires rather structural and governance changes to steer the current economic model to a more circular and sustainable one. The most prominent barriers include: Consumer confusion and lack of trust due to generic sustainability claims and the proliferation of certification schemes and labels; High investment costs and risks for establishing circular bio-based industries; Concerns over (local) biomass feedstock availability within the limits of carrying capacity; Regulatory complexities and lack of support for the transition at the government level; Lack of harmonized waste collection systems and infrastructure and Incompatible and/or insufficient infrastructure capacity for EoL product collection, transportation, storage and management.

**Conclusions:**

Stakeholder involvement is a critical element of the transition to CBBE, as it helps bridge the gap between scientific advancements and practical implementation. This study aims to identify the most prominent barriers to the CBBE transition with an extensive stakeholder engagement strategy with representatives from the construction, chemicals, plastics, and textile sectors. The findings in this study are expected to feed into the further development of policy recommendations to overcome these barriers in the transition.

## 1. Introduction

Recently, there has been a global pursuit of sustainable and resilient economic models. This has prompted a paradigm shift towards a circular bio-based economy (CBBE) at the intersection of circular economy and bioeconomy ^1, 2^. The transition is vital for meeting the United Nations Sustainable Development Goals (SDGs) and reconciling environmental protection with sustainable growth ^3^. Additionally, achieving a net-zero emissions target for the European economy by 2050 requires a shift from a linear fossil-based economy to CBBE ^4^. This transition, at the same time, calls for a new economic framework and policy priorities that are in line with the European Green Deal ^5^. However, this transition is complex and requires societal transformations, cutting-edge technologies, and multi-actor processes.

The shift is exceptionally required in the carbon-intensive sectors facing major challenges within the current linear system, which are responsible for the most significant impacts. The sectors requiring a sustainable transition most urgently are construction, chemicals, plastics, and textiles ^6– 8^. Hence, they can benefit from numerous advantages regarding this transition. For example, the chemicals sector can shift towards a bio-based and circular economy by implementing regulatory measures and providing access to financing mechanisms ^2^. The construction sector can improve sustainability through designing for circularity, deep renovation, and market mechanisms for the widespread reuse of products and materials ^9^. The plastics sector can benefit from European environmental policies and commitments to reduce plastic waste and increase plastic recycling ^10, 11^. Finally, the textile sector can improve its environmental performance and profit margin by reducing the overproduction of fast fashion items and switching to higher-quality garments made from natural fibres ^11, 12^. In general, the transition in all these sectors will require transdisciplinary collaboration among policymakers, educational institutions, industry associations, environmental organisations and public opinion leaders to promote sustainable development.

The active involvement and collaboration of diverse stakeholders across sectors are crucial for the success of CBBE initiatives. This helps to bridge the gap between scientific advancements and practical implementation ^13^. Stakeholder engagement is critical to ensuring the inclusivity, acceptability, and effectiveness of CBBE initiatives. A variety of actors, such as policymakers, industry leaders, research institutions, local communities, and non-governmental organisations, play a key role in shaping the trajectory of CBBE. Stakeholders contribute unique perspectives, local insights, and practical experiences that are invaluable in tailoring CBBE practices to specific contexts and optimising their impact.

The academic literature increasingly explores the importance of stakeholder engagement in identifying solutions to overcome barriers in establishing CBBE ^14^. Some studies emphasise the need for a collaborative and interdisciplinary approach that encompasses multiple sectors and engages stakeholders at various levels ^15^. These studies highlight the multifaceted benefits of stakeholder involvement, ranging from increased resource efficiency and waste reduction to enhanced social acceptance and equitable distribution of benefits ^16^.

Against this background, this paper aims to contribute to the ongoing discourse on CBBE for future research and policy development by synthesising existing knowledge and presenting novel insights as a result of stakeholder engagement activities, i.e., a focus group discussion, expert interviews, and a survey. An extensive list of barriers across four carbon-intensive sectors - construction, chemicals, plastics, and textiles - was initially identified in an extensive grey literature review by Dace *et al.* ^17^ However, stakeholder engagement was considered crucial to validate the barriers identified and their relevance across sectors. This study bridges the gap between the literature and actual CBBE practice by presenting the results of a series of stakeholder engagement activities in which the barriers identified in the literature were discussed, ranked and validated.

The paper is structured as follows: Section 2 provides the literature review on the critical importance of stakeholder engagement in promoting the development, adoption, and optimisation of CBBE strategies and policies. Section 3 presents the methodological basis for the organisation of various stakeholder engagement activities. Section 4 summarises the results and elaborates on the evolution of barriers across various activities. Finally, Section 5 concludes the study with insights and recommendations.

## 2. Literature review

Stakeholder engagement is one of the vital elements in the transition from linear to circular economic models ^14^. Specifically, for the CBBE realm, stakeholders hold the key to fostering collaboration, generating knowledge, and aligning interests towards common goals in shaping policies, practices, and strategies ^18^. Nevertheless, some complexities and challenges related to stakeholder engagement should be taken into consideration.

One of the major complexities lies in the lack of awareness and understanding of the bioeconomy as a whole, its benefits and what roles stakeholders can take within the transition. Several groups, which are key to a thriving European bioeconomy ecosystem, such as the primary sector, youth and financial actors, are currently underrepresented in terms of the level of stakeholder engagement. These categories, which may not yet be fully aware that they could have a role in the bioeconomy, should be involved in the debate and participate in stakeholder engagement activities. Secondly, the perspectives of stakeholders are diverse, with often conflicting interests, values, and motivations ^19^. Therefore, more inclusively and transparently tailored approaches are required to balance out the diversity of stakeholders ^20^. The principle of stakeholder engagement, adopting a "comprehensive" approach, has also been highlighted recently by the European Commission ^19^. Involving stakeholders from diverse fields and backgrounds allows the incorporation of perspectives from various social groups and makes the strategy more forward-thinking and aligned with the needs and agendas of all stakeholders, ensuring that CBBE innovation is relevant, acceptable and finally taken up by them all. Considering the innovations usually occur in “protected spaces”, i.e., *niches*, there are also some stakeholders with concerns about the upscaling of circular solutions ^2^. Additionally, the inherent complexity of circular bioeconomy systems, characterised by interconnectedness and uncertainty, further complicates stakeholder engagement efforts ^21^.

To overcome these challenges and to enhance stakeholder engagement in the CBBE, among others, co-creation strategies have been actively employed. With the help of the co-creation process, e.g., focus groups or participatory workshops, stakeholders collaboratively design and implement solutions, which also lead to fostering ownership and legitimacy ^22^. This collaborative manner creates an environment of open dialogue and knowledge exchange, while facilitating mutual learning among stakeholders ^23^. Consequently, transparent communication builds trust, empowers long-term partnerships and assists in overcoming potential conflicts of interest ^24^.

From a governance point of view, involving stakeholders in the decision-making process, especially through tools like life cycle assessment and multi-criteria analysis, amplifies the robustness and credibility of policy decisions ^25^. Furthermore, ensuring equitable participation and representation of marginalised groups promotes social justice and inclusivity in the circular bioeconomy transition ^21^. Szarka *et al.* ^13^ underline the significance of participatory processes in developing regional bioeconomy strategies that align with local needs and priorities via engaging diverse stakeholders at the regional level. Similarly, Leipold and Petit-Boix ^26^ provide insights into the perspectives of European and German stakeholders on the CBBE, highlighting varying stakeholder interests, priorities, and challenges, underscoring the need for tailored engagement approaches at regional and national levels.

Stakeholder engagement can also be used to understand the market dynamics influencing the transition towards a CBBE and to co-design transition pathways that increase resilience and mitigate potential risks associated with market disruptions ^27^. Ultimately, Lokesh *et al.* ^28^ mention the importance of stakeholder mapping to identify key actors and their roles within bio-based value chains. Engaging stakeholders across the value chain is crucial for addressing gaps, ensuring resource efficiency, and enhancing the viability of CBBE initiatives.

## 3. Methods

This paper presents a follow-up to a comprehensive grey literature review conducted by Dace *et al.* ^17^ as part of the Horizon Europe project SUSTRACK ^29^. The literature analysis was used to look beyond the theoretical background of pure research and to identify more practical solutions from the four sectors of interest (construction, chemicals, plastics, textiles) of the project. A total of 193 different barriers were identified and clustered into six categories: cultural, technical, economic, environmental, governance, and structural. Using statistical methods, we reached a list of 51 barriers to be further validated through co-creation activities.

To that aim, an online focus group entitled “Limits, barriers and solutions to boost the transition towards a circular bio-based economy” was organised during the European Green Week ^30^ ( Section 3.1). Expert interviews were then conducted with the primary objective of validating the previously identified barriers, as well as their allocation to the four selected sectors ( Section 3.2). The results of the interviews were compiled into a new list of barriers to feed an online survey as a final validation step to produce a consolidated list of barriers hindering the transition to CBBE ( Section 3.3).

Written informed consent was obtained from all participants prior to their involvement in the study, in accordance with the SUSTRACK Data Management Plan and Horizon Europe ethical standards. Consent was collected via online registration forms, which also addressed permissions for the use of photographs, video recordings, and other audiovisual materials.

### 3.1 Step 1: Focus group

Focus groups have become a convenient and effective method for collecting qualitative data in a variety of academic and applied research areas ^31^. A focus group (FG) is a group interview that involves a small number of participants with experience in a specific topic of interest. The purpose is to collect qualitative data through interactive and directed discussions with multiple individuals simultaneously ^32, 33^. The participants are encouraged to interact with each other, and group activation is key to exploring and clarifying their perceptions, opinions, and views.

To collect outcomes, the focus group was prepared and structured, adopting the Mobilisation and Mutual Learning approach. According to the methodology developed by the BIOVOICES project ^34^, the starting point is the definition of the *“what for – objectives”.* This step is crucial, as it shapes and guides the three subsequent key questions: *“what – contents to be discussed,” “with whom – stakeholders to be involved,”* and *“how – formats and supporting tools for engagement.”* This approach has been widely validated, having already been applied in more than 300 workshops in the context of EU-funded projects, and was originally developed through the BIOVOICES, Biobridges ^35^ and LIFT ^36^ initiatives. The systematic use of this approach has proven to be effective in structuring participatory processes and ensuring targeted, meaningful dialogue across diverse stakeholder groups.

**Figure 1.**
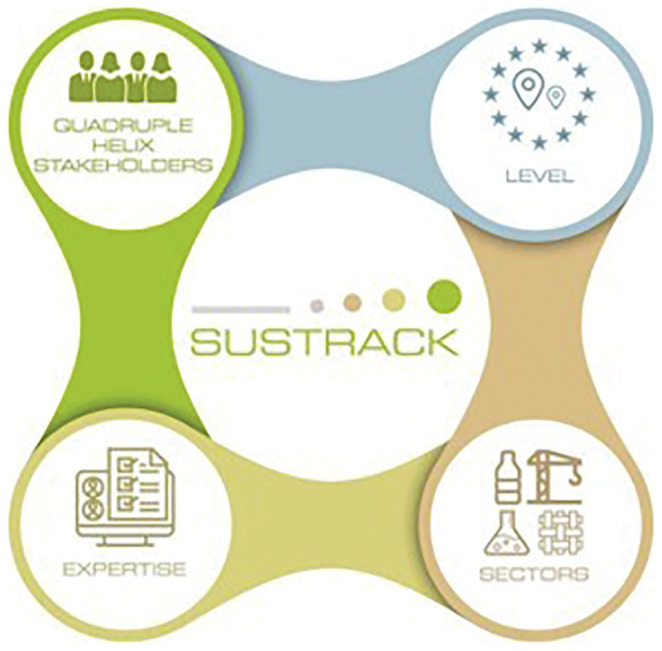
SUSTRACK stakeholder engagement model.

The SUSTRACK stakeholder engagement strategy is based on these experiences and methodological approaches to ensure the engagement of the most appropriate stakeholders to achieve the intended objectives. Prior to the FG, a database of relevant stakeholders was created to ensure the representativeness of the four dimensions defined by the SUSTRACK stakeholder engagement model ( Figure 1), namely the quadruple helix stakeholders, at different geographical levels, sectors of interest, and expertise. The quadruple helix dimension refers to different types of stakeholders (research, business, policy, civil society); the level dimension refers to the geographical scope of stakeholders’ activities (European, national, regional, local); the sector dimension refers to the four SUSTRACK target sectors (textile, construction, chemicals, plastic); and the expertise dimension refers to the relationship between the stakeholder engagement activity and the expected outcomes (e.g., validation, prioritisation, co-creation, foresight).

The FG was organised online, building on the extensive experience gathered during the COVID-19 pandemic, including the fruitful exploitation of online supporting tools such as the MIRO board ^37^. These tools, together with the FG recording, upon appropriate authorisation in compliance with GDPR, facilitated the elaboration of the insights that emerged and significantly reduced the risk of errors in transcribing comments verbatim in real-time.

The FG was structured in four main steps to guide the discussion: (i) setting the scene to facilitate a common ground for discussion; (ii) identification of missing barriers to the transition (compared to the shortlisted list of barriers identified in the literature review); (iii) prioritisation of the most relevant barriers; (iv) allocation of barriers to the four sectors of interest for the SUSTRACK project.

First, the SUSTRACK project was presented to stakeholders, and then each participant had the opportunity to introduce themselves and share their expectations regarding the SUSTRACK project and the event itself. During the FG, a total of 25 external participants were engaged. In the research field, there were 3 stakeholders, each from the construction and plastics sectors, accompanied by one from the chemicals and textiles sectors. The private sector was represented by 3 individuals from construction, 2 from plastics, and one from the chemicals and textiles sectors. NGOs and CSOs had one representative in the construction and plastics sectors. Project-relevant representatives included one stakeholder from the construction, chemicals, and textiles sectors. Additionally, there were participants from other fields, with construction attracting 2, plastics 1, and the chemicals sector 2 people. In total, the construction sector had the highest engagement with 9 participants, followed by the plastics sector with 8, the chemicals sector with 5, and the textiles sector with 3 participants.

**Figure 2.**
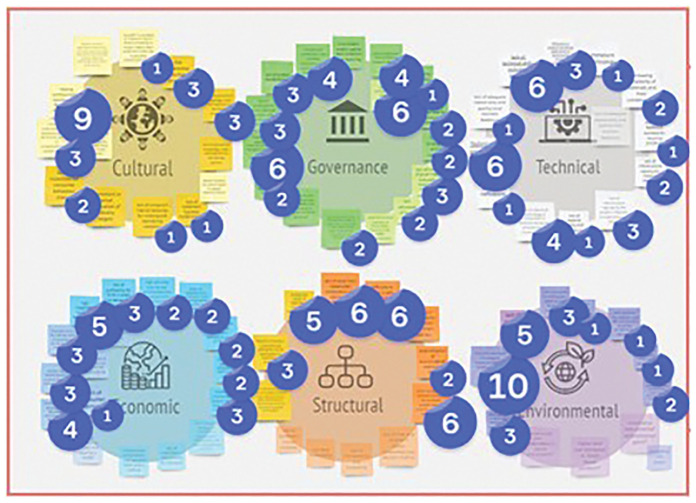
The structure of the MIRO Board for identifying additional barriers and prioritising them.

The results of the barriers identified in the literature review were then presented ^17^. For this step, a total of 54 barriers, including 7 cultural, 11 economic, 4 environmental, 14 governance, 9 structural and 9 technical, were presented for discussion. Then, the interactive session started with the introduction of the online MIRO board, specifically designed to support the process, facilitating the presentation and visualisation of the barriers ( Figure 2). Stakeholders suggested additional barriers based on their background and experience, adding them to the appropriate cluster on the MIRO board.

Stakeholders were then asked to use the voting tool to prioritise the most relevant barriers. They were given 10 votes to select the most urgent barriers to be addressed, based on their opinion ( Figure 2).

**Figure 3.**
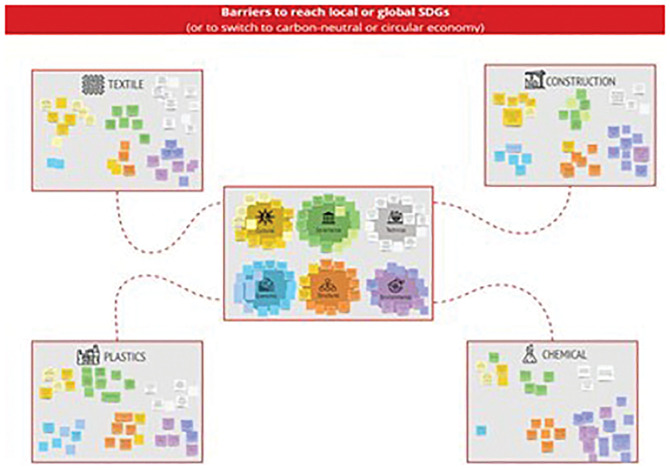
The structure of the MIRO Board to assign barriers to different sectors.

Finally, stakeholders were asked to allocate the barriers to the textile, construction, plastics, and chemicals sectors based on their relevance to each sector ( Figure 3). It should be noted that the same barriers may apply to different sectors.

### 3.2 Step 2: One-to-one interviews with sector experts

**Figure 4.**
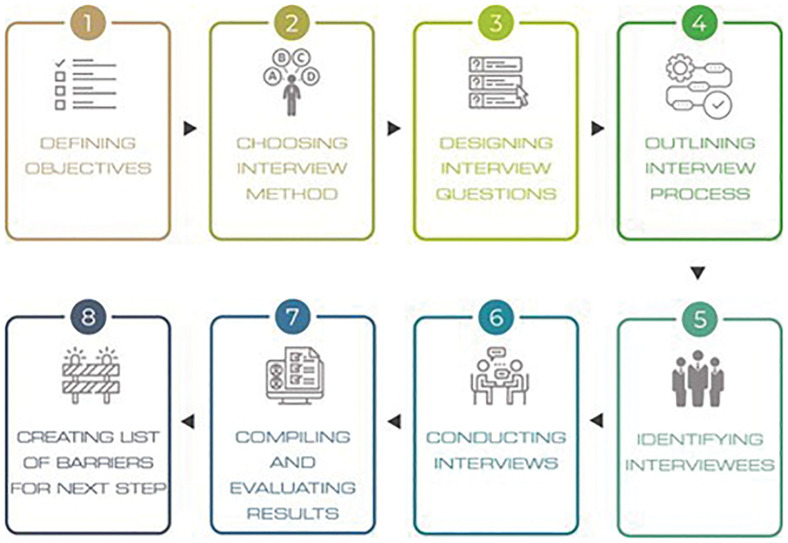
The methodological steps of the interviews.

To further validate and complement the results obtained, 20 expert interviews were conducted online. The interview process included eight steps, presented in Figure 4.

First, the objectives were defined to guide and fit the research objective, namely to validate the identified barriers and allocate them to the four selected sectors. Second, a suitable interview method was selected in line with the aforementioned objective. The semi-structured approach was considered optimal given its versatility, as it accommodates both closed and open-ended questions and allows for follow-up questions if needed ^38, 39^.

**Figure 5.**
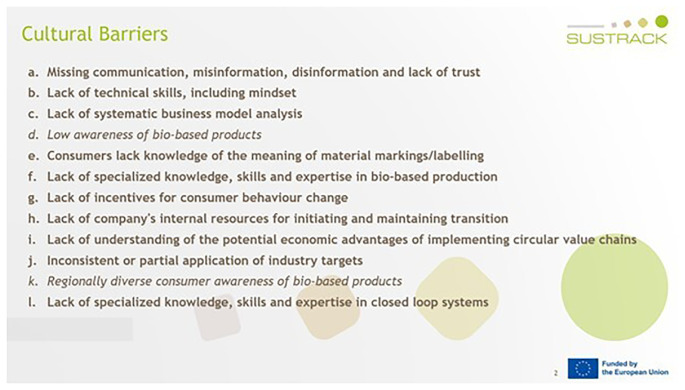
An example PowerPoint slide from an interview for the textile sector, showing how cultural barriers were validated (marked in bold).

The next step was to design the interview questions and compile them into a Microsoft PowerPoint presentation that was shared with the experts during the online interviews ( Figure 5). Microsoft PowerPoint was chosen not only to present the questions in written form to the interviewees, but also to allow the interviewers to take visible notes. This transparency ensured that interviewees were able to review the wording of their responses, enabling them to articulate answers that genuinely reflected their perspectives. Once the interview template had been created, the process was outlined, and potential expert interviewees were identified and contacted. A total of 33 invitations were sent out to prospective experts identified from the SUSTRACK stakeholder database and project networks, and 21 individuals accepted to be interviewed. Participants included representatives from academia (n = 9), industry (n = 7), policy (n = 2) and NGOs (n = 2). Interviews were scheduled for 45–60 minutes and conducted via online meeting platforms. One or two interviewers from the SUSTRACK team conducted each interview using a standardised interview template ( Figure 5). 

The interviewees were asked to validate the barriers previously identified in the literature review and validated during the FG, to allocate them to the sector of their expertise if applicable, and to add any missing barriers ( Figure 5). Once all interviews had been completed, individual reports were created for each interview, and all the findings were analysed and aggregated.

The process of compiling the results of the interviews into a new list of barriers was twofold. On the one hand, the missing barriers suggested by the interviewees were reviewed and compared with the results of the literature review and the FG. Accordingly, some of these barriers were eliminated, and others were used to edit or complement existing barriers. On the other hand, to determine which existing barriers to retain, we applied a pre-defined validation rule: barriers validated by fewer than 40% of interviewed experts were removed from the pre-final list. The 40% threshold was selected *a priori* as a conservative filter to retain items with substantive expert support.

### 3.3 Step 3: Survey

The consolidated list of barriers emerging from the literature review, FG and expert interviews was used to build an online survey implemented in LimeSurvey ^40^. The survey was distributed through the SUSTRACK network, targeted mailing lists, and social media. A total of 55 respondents representing institutions located in nine different European countries completed the survey. Of these, 21 participants (38%) represented one of the four specific sectors targeted by the project. The majority, accounting for 20 participants, represented the bio-based sector as a whole, while the remaining 14 respondents represented “other” sectors such as the blue economy, environment, and recycling. The survey allowed respondents to skip items they did not feel competent to answer.

The survey included a first section collecting respondent characteristics (stakeholder category, country, organisation size and sector represented), followed by the barrier rating blocks categorised into the six clusters (i.e., cultural, economic, environmental, governance, structural, and technical). For each barrier, respondents selected one of four options: “major”, “moderate”, “minor”, or “not a barrier” ( Table 1). A cut-off criterion was applied: barriers deemed major or moderate by at least 75% of survey respondents were retained.

**Table 1.** The description of barrier nature.

Nature of the barrier	Description
Major barrier	A critical obstacle to the transition to a circular bio-based economy. Addressing it requires a coordinated effort and is crucial for the success of the transition
Moderate barrier	A substantial challenge but does not impede the transition as significantly as critical barriers. Addressing it is important, but not inevitable.
Minor barrier	A relatively smaller challenge that may slow down the transition but does not prevent it. While addressing it is not urgent, it should not be ignored.

## 4. Results and discussion

**Figure 6.**
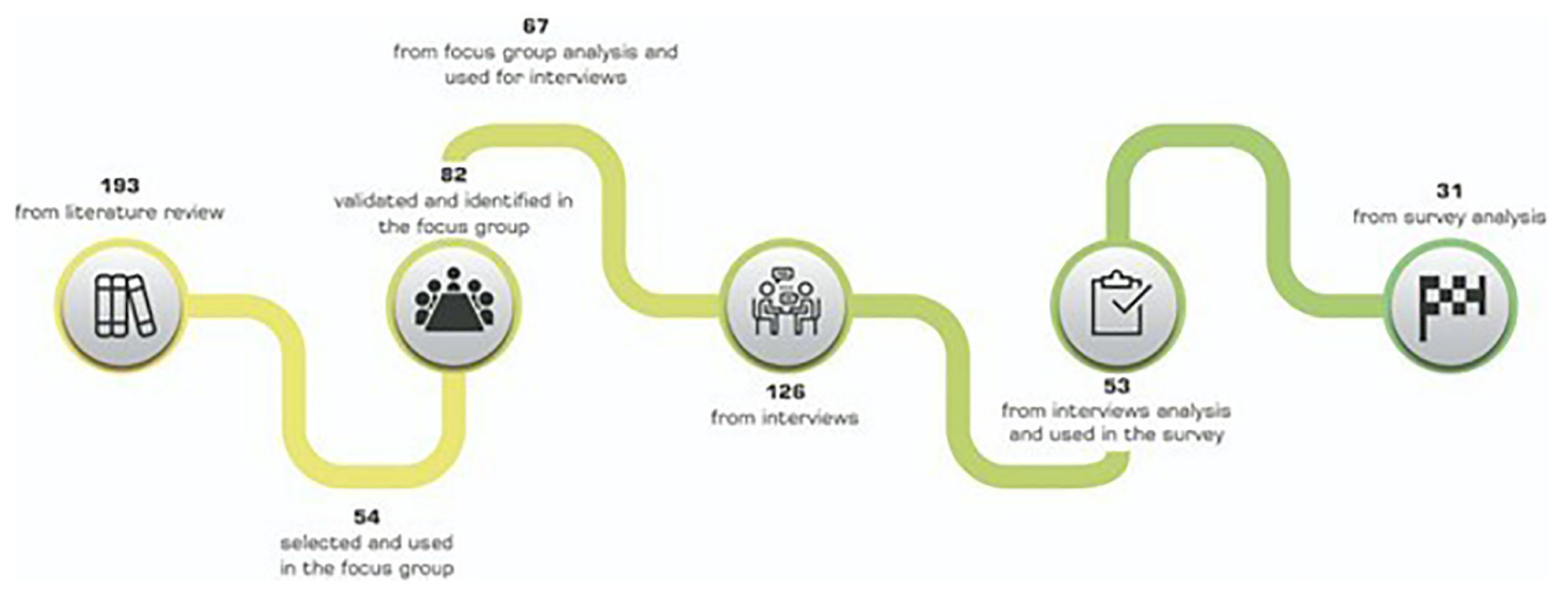
Evolution of barriers across the co-creation activities.

This section outlines the results obtained through different steps of stakeholder engagement activities. A schematic representation of the barrier identification and validation process is shown in Figure 6. The following subsections are dedicated to the findings of different co-creation activities, i.e., Section 4.1 for the focus group results, Section 4.2 for the interview results and Section 4.3 for the survey results.

### 4.1 The results from the focus group

Dace *et al.* ^17^ identified a total of 193 barriers across various sectors, including chemicals (63 barriers), construction (62 barriers), plastics (116 barriers), and textiles (54 barriers). These barriers were mentioned with varying frequencies in the literature. The top three barriers to the transition to a sustainable CBBE were identified as the cultural barrier “Lack of incentives for consumer behaviour change”, which was mentioned in 11 different reports, followed by the structural barrier “Lack of value chain stakeholder collaboration (including information exchange)” (10 reports), and the technical barrier “Increasing complexity of materials and their combinations” (9 reports). These barriers underscore the multifaceted nature of the challenges facing the transition to a sustainable CBBE. To proceed with stakeholder engagement, we applied a cut-off criterion, only considering barriers that were mentioned at least three times in different records. This resulted in a total of 51 barriers, including 14 governance barriers, 11 economic barriers, 9 structural barriers, 7 cultural barriers, and 9 technical barriers. Remarkably, only one barrier was classified under the environmental cluster ^17^. However, underscoring the urgency and importance of addressing environmental concerns, we added three additional environmental barriers from the literature review to initiate the FG discussion on the environmental cluster, despite their initial exclusion based on the cut-off criterion.

During the FG, the list of 54 barriers resulting from the literature review, which served as the starting point for the FG activity, was expanded to 82 barriers. Thus, 28 additional barriers across all clusters were suggested by the participating stakeholders. These 28 barriers included 19 that were identical or similar to ones already identified in the literature review, but did not meet the cut-off criterion and therefore were initially excluded from the presented list. The remaining 9 barriers were considered “original” as they were not previously identified in any of the reviewed literature but rather proposed by the experts participating in the FG.

Following the identification of missing barriers, FG attendees were asked to vote for the 10 most pressing barriers. They were able to allocate their votes to any of the barriers, whether from the provided list or the additional ones they suggested. During the FG, the environmental barrier that received the highest number of votes (10) was the “Lack of harmonised methods for environmental, climate, and sustainability impact assessment of CBBE”, which was suggested during the FG. It highlights the challenge of integrating universally accepted metrics and methodologies to evaluate the sustainability impacts of establishing a CBBE, which leads to uncertainties related to potential benefits. The need for such harmonisation is crucial for providing a reliable basis for monitoring progress towards sustainability goals, thus enhancing transparency. The cultural barrier “Missing communication, misinformation, disinformation and lack of trust” received 9 votes, making it the second most popular choice among stakeholders during the FG. 7 other barriers received 6 votes each, sharing the third place. Of these, 3 are structural, 2 are technical, and 2 are related to governance. Of the 7 barriers, 3 were proposed by stakeholders, while the remaining four came from the list presented ( Table 2).

**Table 2.** Top-rated barriers resulting from the FG.

Rating	Barrier (Votes)	Cluster	Source
1	Lack of harmonised methods for environmental, climate, and sustainability impact assessment of CBBE (10)	Environmental	FG participants
2	Missing communication, misinformation, disinformation and lack of trust (9)	Cultural	FG participants
3	Difficulty to enter a well-established fossil-based market (6)	Structural	Literature review
3	Lack of value chain stakeholder collaboration (including information exchange) (6)	Structural	Literature review
3	Weak reconciliation between the need for economic growth with the need for sustainable development (6)	Structural	Literature review
3	Technical issues for scale-up (6)	Technical	FG participants
3	Lack of technical skills, including mindset (6)	Technical	FG participants
3	Competing political interests with “old” industries (big lobbies, etc.) (6)	Governance	FG participants
3	Fragmented regulatory environment across European countries and at the national/ regional level (6)	Governance	Literature review

It is noteworthy to highlight that only one of the top three most cited barriers in the literature review, specifically the structural barrier “Lack of value chain stakeholder collaboration (including information exchange)”, was prioritised as a top barrier in the FG. The other two most cited barriers, “Lack of incentives for consumer behaviour change” and “Increasing complexity of materials and their combinations”, received only two votes each. Out of the 82 barriers, 31 were voted for by at least three FG participants. Of the remaining barriers, 29 received only one or two votes, and 22 barriers received no votes at all.

The categorisation of the barriers into clusters was undertaken to find structure within the long list of barriers and group similar barriers together, separating them from those that are dissimilar. It is important to acknowledge that such classificatory activities are carried out on a subjective level, and there might be some overlap between categories. However, these clusters still provide a useful framework for analysis. As demonstrated in the table above, none of the top-rated barriers is economic. Of the three top-rated structural barriers, however, two can be viewed as economic, namely “Difficulty to enter a well-established fossil-based market” and “Weak reconciliation between the need for economic growth with the need for sustainable development”. A reallocation of the barriers to different clusters can be beneficial in revealing underlying factors. For instance, “Difficulty entering a well-established fossil-based market” could be seen as both a structural barrier due to the dominance of existing players and an economic barrier due to the lack of economic incentives for new entrants.

After voting, stakeholders were asked to allocate the barriers to their respective sectors of interest. Of the barriers, 31 were allocated to the plastics sector, 29 were relevant to both the textiles and construction sectors, and 25 were relevant to the chemicals sector. Only 3 barriers were allocated to all four sectors, whereas 21 were not allocated to any sector at all. Despite this, some of these barriers still received votes, including the technical barrier “Lack of technical skills, including mindset”, which shared third place in the prioritisation exercise. The 3 common barriers allocated to all sectors are: (i) Missing communication, misinformation, disinformation, and lack of trust; (ii) Lack of harmonised methods for environmental, climate, and sustainability impact assessment of CBBE; and (iii) Weak reconciliation between the need for economic growth with the need for sustainable development.

As the final step, we formulated a consolidated list of all barriers based on the feedback collected during the barrier rephrasing process and the addition of newly identified barriers. Overlapping barriers were merged, resulting in a concise list of 67 barriers for the follow-up project activities (Appendix A ^41^). No barriers were removed from the originally provided list of 54 barriers.

### 4.2 The results from expert interviews

The resulting list of 67 barriers after FG was validated by experts during interviews and allocated to four key sectors: textiles, construction, plastics, and chemicals. The most frequently cited barrier across sectors was “Missing communication, misinformation, disinformation, and lack of trust,” while “Lack of systematic business model analysis” was the least cited.

For the textiles and plastics sectors, all 67 barriers were validated by at least one expert, though strong consensus was limited. In the textiles sector, 10 barriers were unanimously validated, whereas the plastics sector achieved unanimous agreement on only one: the environmental barrier “Lack of harmonised methods for environmental, climate, and sustainability impact assessment of CBBE.” In contrast, chemicals sector experts demonstrated higher consensus, unanimously validating 14 barriers, half of which fell under governance. However, two barriers, “Lack of systematic business model analysis” (cultural) and “Insufficient investment in mature closed-loop recycling technologies” (economic), received no validation from chemicals sector experts. The construction sector exhibited the greatest number of unvalidated barriers (10), likely due to the limited representation of experts (n=3).

During the interviews, participants identified 59 additional barriers, expanding the list to 126. These new barriers spanned the six clusters: cultural (14), economic (12), governance (12), structural (8), environmental (7), and technical (6). Many new barriers were duplicates, closely related, or not true barriers according to our definition of a barrier, i.e. “a problem, rule or situation that prevents somebody from doing something, or that makes something impossible” ^42^.

The process of compiling the results of the interviews into a new list of barriers was twofold. On the one hand, newly proposed barriers were compared against the literature findings of Dace *et al.* ^17^, leading to the elimination or refinement of some barriers. On the other hand, existing barriers validated by less than 40% of the experts were removed.

The process culminated in a pre-final list of 53 barriers. Although some edits were made for clarity and precision, the number of barriers remained unchanged, and it is presented in Appendix A.

### 4.3 The final list of barriers

The 53 barriers resulting from the interview step were prioritised through a survey, with the objective of identifying those that most significantly hinder the transition to a CBBE. Survey respondents rated barriers as major, moderate, minor, or non-barriers ( Table 1). A cut-off criterion was applied: barriers deemed major or moderate by at least 75% of survey respondents were retained. This process reduced the list to 31 key barriers ( Table 3). Notably, four barriers were identified as major by at least half of the respondents (28 out of 55), three of which belonged to governance, while one was an economic barrier. Detailed survey results are provided in Appendix B ^41^.

**Table 3.** The final list of validated barriers.

Cluster	Barrier
Cultural	Missing communication, misinformation, and disinformation
	Consumer confusion and lack of trust due to generic sustainability claims and the proliferation of certification schemes and labels
	Low awareness and knowledge of bio-based products
	Lack of incentives for consumer behaviour change
Economic	High investment costs and risks for establishing circular bio-based industries
	Fluctuating prices and unstable supply of biomass and (processed) raw materials
	Weak cost competitiveness: bio-based vs. fossil-based ingredients, materials or products
	Difficulties in reconciling economic growth with sustainable development
	High operating costs: bio-based vs. fossil-based production
	Lack of funding and investment in End-of-Life (EoL) infrastructure and waste management practices
Environmental	Concerns over (local) biomass feedstock availability within the limits of carrying capacity
	Lack of harmonised methods for environmental, climate and sustainability impact assessments
	Lack of knowledge and data on impacts
Governance	Regulatory complexities and lack of support for the transition at government level
	Fragmented regulatory framework across European countries and at national/ regional level
	Competing political interests with fossil-based industries (big lobbies etc.)
	Lack of alignment and consistency between different policies
	Lack of market-based policies (e.g. subsidies or higher CO2 prices) to stimulate the market
	Limited long-term policy reliability
	Lack of supportive policy framework for product-related inner circles of the circular system (recycling, reuse, repair, redistribute, remanufacture, and refurbish)
	Legislative limitations for applications of materials when classified as waste
	Lack of clarity around policy targets and trajectories
	Insufficiently ambitious targets and limited policy drivers
Structural	Lack of harmonized waste collection systems and infrastructure
	Lack of established markets and value chains for by-products
	Low data accessibility and lack of transparency and traceability
	Lack of value chain stakeholder collaboration (including information exchange)
	Social and technical lock-ins to the current linear system
	Lack of mid- and long-term impact assessment and decision-making
Technical	Incompatible and/or insufficient infrastructure capacity for EoL product collection, transportation, storage and management
	Increasing complexity of materials and their combinations

The results of the multi-stakeholder consultation have shown that the primary cultural barrier is the lack of communication regarding the advantages of bio-based products. This is closely followed by consumer confusion and mistrust arising from ambiguous sustainability claims. As a result of these barriers, there remains a limited understanding of bio-based products. Additionally, a significant challenge hindering the transition is the absence of incentives to encourage changes in consumer behaviour.

The most challenging barrier within the economic cluster is the high costs and risks involved in setting up circular bio-based systems. Furthermore, bio-based production also incurs higher operating costs.

The most pressing environmental challenge is the concern of (local) biomass within the limits of the carrying capacity. Additionally, the lack of knowledge and data availability for assessing the impact on the environment of the different steps of the life cycle of bio-based products, coupled with the lack of harmonised methods for environmental, climate, and sustainability impact assessment, are seen as urgent concerns to be addressed.

The most challenging governance barriers are related to the lack of a supportive regulatory framework for establishing a circular bio-based economy, characterised by complexity and conflicting policy goals. Additionally, the lack of a level playing field and the existence of competing political interests between the fossil-based and the bio-based economy are emphasised.

Within the structural cluster, the most pressing barrier is the lack of harmonised waste collection systems and infrastructure. In addition, the lack of established markets and value chains for byproducts is hampering the establishment of circular business models, and this is related to the technical lock-ins to the current linear system. Experts also identified the collaboration among value chain stakeholders as a challenge to be overcome to support the establishment of a circular bio-based economy.

Immature technologies and insufficient capacities for EoL product treatments are considered the most challenging barriers within the technical cluster. In addition, the increasing complexities of materials and their combinations were also viewed as a major challenge, which in turn can affect both the production and the EoL treatment of these products.

The list of 31 validated barriers indicates that the transition is asking for rather structural and governance changes to steer the current economic model to a more circular and sustainable one. In this study, we highlighted the importance of adopting a joint approach combining literature review and stakeholder engagement activities to improve the understanding of the problem and detect the hotspots to be taken into account in developing solution strategies and policy recommendations.

## 5. Conclusion

In the pursuit of sustainable and resilient economic models, a paradigm shift towards a CBBE has emerged as an innovative approach. Stakeholder involvement is a critical element of this transition to CBBE, as it helps bridge the gap between scientific advancements and practical implementation. This study aims to identify the most prominent barriers to the CBBE transition with an extensive stakeholder engagement strategy with representatives from the construction, chemicals, plastics, and textile sectors.

Upon a series of activities, adopting the SUSTRACK stakeholder engagement strategy, we reached a list of 31 barriers that are hindering the transition to a CBBE. In summary, the most prominent barriers in each cluster include (i) Consumer confusion and lack of trust due to generic sustainability claims and the proliferation of certification schemes and labels; (ii) High investment costs and risks for establishing circular bio-based industries; (iii) Concerns over (local) biomass feedstock availability within the limits of carrying capacity; (iv) Regulatory complexities and lack of support for the transition at the government level; (v) Lack of harmonized waste collection systems and infrastructure and (vi) Incompatible and/or insufficient infrastructure capacity for EoL product collection, transportation, storage and management.

The findings in this study are expected to feed into the further development of policy recommendations to overcome these barriers in the transition. To this aim, follow-up studies are planned to make a prioritisation of the list of barriers across different sectors and provide tailor-made policy options to remove the barriers.

## Ethical and consent

The SUSTRACK project (funded under the Horizon Europe programme Grant Agreement number 101081823) received Ethics Clearance from the European Commission following an Ethics Check at the proposal stage (May 2022). The project Data Management Plan, prepared in accordance with the European Commission’s recommended structure and including ethics considerations, ensures compliance with the EU General Data Protection Regulation (GDPR) and relevant national regulations. Since the European Commission’s Ethics Clearance is deemed sufficient for Horizon Europe projects, no additional approval from any institutional review board was obtained.

The informed consent procedure is defined in the SUSTRACK Data Management Plan and complies with the standards of the Horizon Europe programme. Written informed consent was obtained from all participants prior to their involvement in project activities. Consent was collected through online registration forms (via third-party provider within secure environment), which included specific sections covering participation in workshops and events, as well as the use of photographs, video recordings, and other audiovisual materials. Participants were informed about the purpose of data collection, the scope of data use, and their rights under the EU General Data Protection Regulation (GDPR). Only individuals who provided explicit consent were included in the study.

## Data Availability

Zenodo: Dataset from the SUSTRACK project, available at: https://zenodo.org/records/17610017 ^41^ The dataset includes: Appendix A. Evolution of barriers for review.xlsx (spreadsheets showing how the identified barriers under each cluster were changed during the various stakeholder engagement activities). Appendix B. Ranking of barriers for review.xlsx (spreadsheets showing the survey results of how the identified barriers under each cluster were ranked). Survey questions Interview presentation materials Supporting forms and templates (informed consent form and data subject form) Data are available under the terms of the Creative Commons Zero "No rights reserved" data waiver (CC0 1.0 Public domain dedication).
