# Supplementary material for: Insights from an extensive multi-step multi-stakeholder engagement in circular bio-based economy
Source: Open Res Eur. 2026 May 9;5:389. Originally published 2025 Dec 17. [Version 2] doi: 10.12688/openreseurope.21512.2 (PMC13197747; doi:10.12688/openreseurope.21512.2)
Supplement: Peer review history

# Reviewer report: Reviewer response for version 2

**DOI:** 10.21956/openreseurope.25744.r73891

**Published:** 2026-05-22

**Authors:** Dinh Linh Le

**Affiliations:** 1 Circular Economy Solutions Unit, The Finnish Environment Institute, -, Finland

Many thanks for addressing my comments in the first round. I am now happy with the revisions.

If applicable, is the statistical analysis and its interpretation appropriate?

Partly

Is the study design appropriate and is the work technically sound?

Partly

Is the work clearly and accurately presented and does it engage with the current literature?

Partly

Are the conclusions drawn adequately supported by the results?

Partly

Are sufficient details of methods and analysis provided to allow replication by others?

Yes

Are all the source data and materials underlying the results available?

Yes

Reviewer Expertise:

I am a senior researcher in circular economy in the construction industry at the Finnish Environment Institute. My research areas are circular construction, sustainable construction, and sustainability assessments in the built environment.

I confirm that I have read this submission and believe that I have an appropriate level of expertise to confirm that it is of an acceptable scientific standard.

# Reviewer report: Reviewer response for version 2

**DOI:** 10.21956/openreseurope.25744.r73890

**Published:** 2026-05-13

**Authors:** George Banias

**Affiliations:** 1 Centre for Research and Technology-Hellas, Thessaloniki, Greece

*The authors have addressed all previously raised queries, and I have no further comments to add. I approve this version.*

If applicable, is the statistical analysis and its interpretation appropriate?

Yes

Is the study design appropriate and is the work technically sound?

Yes

Is the work clearly and accurately presented and does it engage with the current literature?

Yes

Are the conclusions drawn adequately supported by the results?

Yes

Are sufficient details of methods and analysis provided to allow replication by others?

Yes

Are all the source data and materials underlying the results available?

Yes

Reviewer Expertise:

Environmental Engineering, Circular (Bio-)economy and sustainability, circularity and sustainability assessment measurement, DPP, biomass utilisation,

I confirm that I have read this submission and believe that I have an appropriate level of expertise to confirm that it is of an acceptable scientific standard.

# Reviewer report: Reviewer response for version 1

**DOI:** 10.21956/openreseurope.23268.r66503

**Published:** 2026-01-07

**Authors:** Dinh Linh Le

**Affiliations:** 1 Circular Economy Solutions Unit, The Finnish Environment Institute, -, Finland

Transitioning to a circular bioeconomy could be considered one of the best solutions to reduce reliance on virgin materials and support sustainable development. The paper presents some interesting results, but it still needs substantial revision. Please see my comments below for more detail.

  

- In the second paragraph of the Introduction, when mentioning the construction industry, the authors should also discuss the use of bio-based, and especially circular bio-based building materials. There are studies focused on the use of these materials for supporting the transition of the economy to circular (bio)economy, which might be helpful for the authors.
- The authors have not provided a comprehensive background on similar existing studies and then critiqued their shortcomings to justify the need for this paper. Do any studies in this domain exist? How does the present paper differ from them?
- Since barriers to the transition to a circular bioeconomy are sector-specific, why do the authors group them together as “umbrella” barriers for the whole economy? Does this approach make sense —especially given that, in the Conclusions, the authors state they will conduct follow-up studies focusing on specific sectors?
- Section 2 (Literature Review) should be combined with the Introduction to better provide background information and strengthen the rationale for conducting this study. Otherwise, it needs to be deepened.
- Regarding the Methods section, it would be helpful if the authors could provide a comprehensive figure illustrating all research steps. This might improve the logic and flow of the paper. It is also unclear where and how the authors identified the original list of 193 barriers. Are these from a previous study? The authors should clearly explain this and specify the methods used to analyse the data (both qualitative and quantitative).
- The authors should deepen the Discussion by benchmarking their findings against existing studies in the field.
- The implications of the research are underdeveloped. This is important to highlight the value and relevance of the study.
- The Conclusion heading should be “Conclusions” (plural). In this section, the authors should also indicate the research limitations and outline how these limitations could be addressed in future work. The implications of the research should also be briefly reiterated.

If applicable, is the statistical analysis and its interpretation appropriate?

Partly

Is the study design appropriate and is the work technically sound?

Partly

Is the work clearly and accurately presented and does it engage with the current literature?

Partly

Are the conclusions drawn adequately supported by the results?

Partly

Are sufficient details of methods and analysis provided to allow replication by others?

Yes

Are all the source data and materials underlying the results available?

Yes

Reviewer Expertise:

I am a senior researcher in circular economy in the construction industry at the Finnish Environment Institute. My research areas are circular construction, sustainable construction, and sustainability assessments in the built environment.

I confirm that I have read this submission and believe that I have an appropriate level of expertise to state that I do not consider it to be of an acceptable scientific standard, for reasons outlined above.

# Response

**Published:** 2026-04-23

**Authors:** Gülşah Yilan

**Affiliations:** Law and Economics, University of Rome Unitelma Sapienza, Rome, Italy

**Comment from the Reviewer**

**Response**

 1 In the second paragraph of the Introduction, when mentioning the construction industry, the authors should also discuss the use of bio-based, and especially circular bio-based building materials. There are studies focused on the use  of these materials for supporting the transition of the economy to circular (bio)economy, which might be helpful for the authors.

*The authors added the reference to a recent work highlighting the importance of the use of circular bio-based building materials to support the transition.*

 2 The authors have not provided a comprehensive background on similar existing studies and then critiqued their shortcomings to justify the need for this paper. Do any studies in this domain exist? How does the present paper differ from them?

*The authors enriched the literature review section by providing an overview of similar studies and underlining their limitations in stakeholder engagement in the transition to CBBE, as well as a clear description of how this paper is different from the literature.*

 3 Since barriers to the transition to a circular bioeconomy are sector-specific, why do the authors group them together as “ **umbrella**” barriers for the whole economy? Does this approach make sense —especially given that, in the Conclusions, the authors state they will conduct follow-up studies focusing on specific sectors?

*This paper was structured to be the first step of the stakeholder engagement and barrier screening process. For this reason, sector-specific insights were provided to a limited extent, but not in detail. However, in the project, AHP and ANP methods have been implemented as the follow-up steps for better understanding the sectoral dynamics. That is in the scope of another paper in progress by the authors, and a clear reference has been added to the upcoming study in the conclusions. This limitation has also been mentioned in the conclusions section.*

 4 Section 2 (Literature Review) should be combined with the Introduction to better provide background information and strengthen the rationale for conducting this study. Otherwise, it needs to be deepened.

*The authors decided to deepen Section 2 with specific references to similar studies. Please see comment #2.*

 5 Regarding the Methods section, it would be helpful if the authors could provide a comprehensive figure illustrating all research steps. This might improve the logic and flow of the paper.   It is also unclear where and how the authors identified the original list of 193 barriers. Are these from a previous study? The authors should clearly explain this and specify the methods used to analyse the data (both qualitative and quantitative).

*In the previous version, Figure 6 has been provided to summarise the method and the evolution of the barriers. A revised figure has been inserted, providing more details from the methods applied to enhance readability.   Yes, the original list was already published in another work by the same project. A clear reference has been provided to the work performed before, also underlining the methods used to analyse the data.*

 6 The authors should **deepen the Discussion by benchmarking their findings against existing studies in the field**.

*The authors provided more reflection on the discussion on findings in comparison with the previously published studies, under section 4.3.*

 7 The **implications** of the research are underdeveloped. This is important to highlight the value and relevance of the study.

*The authors added a new subsection regarding the implications on policy, practice and future research (Section 4.4)*

 8 The Conclusion heading should be “Conclusions” (plural). In this section, the authors should also indicate the research limitations and outline how these limitations could be addressed in future work. The **implications** of the research should also be briefly reiterated.

*The heading has been corrected. The research limitations have been indicated. The implications were also briefly mentioned in the conclusions.*

# Reviewer report: Reviewer response for version 1

**DOI:** 10.21956/openreseurope.23268.r66496

**Published:** 2025-12-29

**Authors:** George Banias

**Affiliations:** 1 Centre for Research and Technology-Hellas, Thessaloniki, Greece

Reading the title and having a first glance at the abstract, it initially appeared to be a promising manuscript. The paper addresses a timely and policy-relevant topic in the domain of sustainability transitions, focusing on barriers to the transition towards a circular bio-based economy (CBBE). The manuscript reports the results of a multi-step stakeholder engagement process (focus group, expert interviews, and survey) aimed at discussing, ranking, and validating barriers initially identified through an extensive grey literature review. I have carefully read the article.

 The topic is well aligned with the scope of Open Research Europe, and the paper may be of interest to its readership.

 The manuscript’s main strength lies in its systematic triangulation of evidence across three engagement steps and multiple stakeholder groups and sectors (construction, chemicals, plastics, textiles). The process is clearly described, and the final output—a consolidated list of 31 validated barriers across six clusters (cultural, economic, environmental, governance, structural, technical)—is relevant and potentially useful for subsequent policy work. However, several aspects require clarification and methodological strengthening to ensure that the reported findings and analytical choices are fully transparent and that conclusions are appropriately supported. I therefore recommend Approved with Reservations.

 I hope the following detailed comments will help the authors improve their work. My main objections for indexing the paper in its current form are the following:

- The interview-stage exclusion rule (barriers validated by fewer than 40% of experts removed) and the survey cut-off (≥75% “major/moderate”) are stated, but the rationale remains limited. A short sensitivity reflection would increase confidence that results are not overly threshold-dependent.
- The construction sector has limited expert interview representation (n=3), which the manuscript notes. However, the likely impact on sectoral comparability and on which barriers survive the 40% rule should be discussed more explicitly. 
- The results would benefit from deeper interpretation of why specific barriers dominate, and how barriers interact across clusters (e.g., governance–economic linkages; structural lock-ins shaping technical constraints). This would strengthen the contribution beyond a catalogue of barriers. 
- The manuscript describes how barriers expand (e.g., 54→82 in the focus group; 67 validated in interviews; then reduced to 53 and finally 31), but it would help readers if the logic of merging/rephrasing and the criteria for removing “duplicates / not true barriers” were summarised more systematically in the main text (even if details remain in appendices). 
- Within the Methods section mention using “statistical methods” to reduce 193 to 51 barriers prior to validation, but the manuscript does not specify the exact procedure (e.g., frequency analysis only; weighting by sector; handling of multi-sector mentions). The authors should enhance this step to be more explicit. 
- The study uses networks and stakeholder databases; this is reasonable, but a concise discussion of selection bias risk (e.g., over-representation of actors already engaged in CBBE initiatives) and its implications for generalizability would strengthen validity. 
- Statements such as the transition “requires rather structural and governance changes” are plausible and aligned with the prioritised barriers, but the authors should ensure that such general claims are framed as supported by the stakeholder validation results (and not as broader causal inference). 
- Although several figures are included (e.g., interview steps and barrier evolution), the authors should add a single concise schematic that shows the full pipeline (193→51→FG expansion→interview validation→survey cut-off→31) with sample sizes and decision rules would improve accessibility for readers. 
- Some barriers are phrased slightly differently across sections (e.g., communication/trust-related cultural barriers). The authors should harmonise the terms between the narrative and Table 3 would prevent ambiguity. 

If applicable, is the statistical analysis and its interpretation appropriate?

Yes

Is the study design appropriate and is the work technically sound?

Yes

Is the work clearly and accurately presented and does it engage with the current literature?

Yes

Are the conclusions drawn adequately supported by the results?

Yes

Are sufficient details of methods and analysis provided to allow replication by others?

Yes

Are all the source data and materials underlying the results available?

Yes

Reviewer Expertise:

Environmental Engineering, Circular (Bio-)economy and sustainability, circularity and sustainability assessment measurement, DPP, biomass utilisation,

I confirm that I have read this submission and believe that I have an appropriate level of expertise to confirm that it is of an acceptable scientific standard, however I have significant reservations, as outlined above.

# Response

**Published:** 2026-04-23

**Authors:** Gülşah Yilan

**Affiliations:** Law and Economics, University of Rome Unitelma Sapienza, Rome, Italy

**Comment from the Reviewer** **Response**

 1 The interview-stage exclusion rule (barriers validated by fewer than 40% of experts removed) and the survey cut-off (≥75% “major/moderate”) are stated, but the rationale remains limited. A short sensitivity reflection would increase confidence that results are not overly threshold-dependent. The rationale for selecting 40% and 75% for the interview phase and the survey phase, respectively, has been provided in Sections 3.2 and 3.3 with a scientific reference to the survey respondents as well.

 2 The construction sector has limited expert interview representation (n=3), which the manuscript notes. However, the likely impact on sectoral comparability and on which barriers survive the 40% rule should be discussed more explicitly. This is also one of the reasons we preferred expressing the results under an overarching approach rather than providing deep sector-specific analysis. We highlighted this limitation in the conclusions section.

 3 The results would benefit from deeper interpretation of why specific barriers dominate, and how barriers interact across clusters (e.g., governance–economic linkages; structural lockins shaping technical constraints). This would strengthen the contribution beyond a catalogue of barriers. More insight has been provided under the discussion of the final list of barriers in Section 4.3.

 4 The manuscript describes how barriers expand (e.g., 54→82 in the focus group; 67 validated in interviews; then reduced to 53 and finally 31), but it would help readers if the logic of merging/rephrasing and the criteria for removing “duplicates / not true barriers” were summarised more systematically in the main text (even if details remain in appendices). We provided examples of barrier evolution, considering two barriers

- “Consumers lack of knowledge of the meaning of material markings/labelling” has been reworded as “Consumer confusion and lack of trust due to generic sustainability claims and the proliferation of certification schemes and labels”
- “Missing communication, misinformation, disinformation and lack of trust” has been reworded to “Missing communication, misinformation, and disinformation”

5 Within the Methods section mention using “statistical methods” to reduce 193 to 51 barriers prior to validation, but the manuscript does not specify the exact procedure (e.g., frequency analysis only; weighting by sector; handling of multi-sector mentions). The authors should enhance this step to be more explicit. The authors provided a specific reference to their earlier study where they applied the statistical method, along with a citation of the method used.

 6 The study uses networks and stakeholder databases; this is reasonable, but a concise discussion of **selection bias risk** (e.g., over-representation of actors already engaged in CBBE initiatives) and its implications for generalizability would strengthen validity. We agree that selection bias is a risk in stakeholder-led research. In our revision, we have added a concise discussion on this in Section 3 (Methods) and Section 5 (Conclusions). Specifically, we acknowledge that our sample may lean toward actors already active in the CBBE transition. However, to mitigate this, we employed a multi-dimensional stakeholder model. Furthermore, our 3-step validation process utilised a conservative 75% consensus threshold for the final barrier list. This ensures that the results represent broad, cross-sectoral challenges rather than the specialised interests of a small niche. While this strengthens the validity for these specific carbon-intensive sectors, we have added a note on the limitations of generalizability to actors entirely outside the current bioeconomy ecosystem.

 7 Statements such as the transition “requires rather structural and governance changes” are plausible and aligned with the prioritised barriers, but the authors should ensure that such general claims are framed as supported by the stakeholder validation results (and not as broader causal inference). The statement has been revised as “The literature findings validated through stakeholder engagement underline that the transition requires rather structural and governance changes to steer the current economic model to a more circular and sustainable one”

 8 Although several figures are included (e.g., interview steps and barrier evolution), the authors should add a single concise schematic that shows the full pipeline (193→51→FG expansion→interview validation→survey cut-off→31) with sample sizes and decision rules would improve accessibility for readers. In the previous version, Figure 6 has been provided to summarise the method and the evolution of the barriers. A revised figure has been inserted, providing more details from the methods applied to enhance readability.  

 9 Some barriers are phrased slightly differently across sections (e.g., communication/trust-related cultural barriers). The authors should harmonise the terms between the narrative and Table 3 to prevent ambiguity. This is mainly due to the revision of barriers along the 3-step methodology. It is also provided as an explicit example how the barrier “Missing communication, misinformation, disinformation and lack of trust” (in Table 2) has been reworded after the interviews and included in the final list as “Missing communication, misinformation, and disinformation” while the part related to trust was included in another barrier as “Consumer confusion and lack of trust due to generic sustainability claims and the proliferation of certification schemes and labels” (Table 3). We also put a note on Table 2 to alert the reader about any discrepancies across tables.
